# Longitudinal Changes in Subjective Well‐Being and Loneliness in Parkinson's Disease and Focal/Segmental Craniocervical Dystonia

**DOI:** 10.1002/mdc3.70742

**Published:** 2026-08-03

**Authors:** Suzette Shahmoon, Dejan Georgiev, Paul Jarman, Kailash Bhatia, Patricia Limousin, Marjan Jahanshahi

**Affiliations:** ^1^ Department of Clinical and Movement Neurosciences UCL Queen Square Institute of Neurology London UK; ^2^ Department of Neurology University Medical Centre Ljubljana Ljubljana Slovenia; ^3^ Faculty of Computer and Information Sciences University of Ljubljana Ljubljana Slovenia

**Keywords:** focal/segmental cervical dystonia, happiness, hope, life satisfaction, loneliness, meaning in life, Parkinson's disease, subjective well‐being

## Abstract

**Background:**

The longitudinal changes of subjective well‐being (SWB) in Parkinson's disease (PD) and focal/segmental craniocervical dystonia (FSCD) are poorly understood.

**Methods:**

Fifty‐three people with PD and 41 with FSCD completed questionnaires assessing various aspects of SWB (subjective happiness, hope, meaning in life, life satisfaction) and loneliness at baseline and one year later.

**Results:**

Subjective happiness, life satisfaction and loneliness did not change over the one year follow‐up period. Hope agency decreased in PD (*P* < 0.001) and increased in FSCD (*P* = 0.026). Presence of meaning in life increased for FSCD (*P* = 0.021), accompanied by a decrease in search for meaning in life in this group (*P* = 0.041). Both domains remained stable in PD.

**Conclusions:**

In PD and FSCD, SWB and loneliness were generally stable over one year, but there were disease‐specific changes in certain domains of SWB (hope, meaning in life). Further longitudinal studies with longer follow‐up periods are needed.

Subjective well‐being (SWB) is a complex construct encompassing subjective happiness, hope, meaning in life, and life satisfaction.^1^, ^2^ Loneliness is defined as a state in which an individual feels socially isolated due to deficiencies in the quantity or quality of social interactions.^3^ Recent evidence indicates differences in subjective well‐being (SWB) and loneliness between people with Parkinson's disease (PwPD) and people with focal/segmental craniocervical dystonia (PwFSCD).^2^, ^3^ Compared to healthy controls (HC), PwPD and PwFSCD reported significantly lower life satisfaction and meaning in life, but sought meaning in life more than HCs. Moreover, PwFSCD felt lonelier than HCs, but there were no differences in loneliness between PwPD and HCs or PwPD and PwFSCD.^3^

Evidence on the longitudinal changes of SWB and loneliness in PwPD and PwFSCD is scarce. A decline in life satisfaction over three years, predicted by walking difficulties and general self‐efficacy, has been demonstrated.^4^ In FSCD mood and health‐related quality of life often remain stable in short‐term follow‐ups.^5^ Therefore, we set out to explore the longitudinal changes in SWB and loneliness in PwPD and PwFSCD.

## Methods

### Study Design

This was a longitudinal, one year follow‐up comparative study on SWB and loneliness in PwPD and PwFSCD.^2^, ^3^

### Participants

Fifty‐nine PwPD and 49 PwFSCD agreed to participate in this study. Fifty‐three PwPD (response rate 89%) and 41 PwFSCD (response rate 84%) finally accepted the invitation. The method used to determine the sample size is described elsewhere.^2^, ^3^ People with PD and PwFSCD were recruited through the outpatient clinics of three clinicians (PL, KB, and PJ) working at the National Hospital of Neurology and Neurosurgery, Queen Square, London. Eligible participants emailed the research team, and a questionnaire pack was sent to them. All participants were assessed twice, one year apart. A booklet containing the scales and questionnaires listed below, along with an information sheet explaining the aims of the study, was mailed to each potential participant. As suggested by the Ethics Committee, who reviewed and approved the proposal, the return of the completed booklet constituted implied consent. Ethical approval was granted by the Health Research Authority and the Health and Care Research Wales Ethics Committee. Full ethical approval for the project was obtained on February 2, 2018, application number 233401, REC Ref 18/LO/1368.

In addition to demographic information on age, gender, marital status (married, single, separate, divorced, widowed, having a partner), employment (retired, unemployed, full time employment, part time employment), (note that information on educational level, racial and ethnic identity was not collected), the following validated and standardized scales and questionnaires were used to assess participants’ subjective and mental well‐being (for a detailed description, see Supporting Information S1):

- *Measures of subjective wellbeing: Subjective Happiness Scale* (SHS),^6^ *State Hope Scale* (SHoS),^7^ *Meaning in Life Questionnaire* (MLQ),^8^ *Satisfaction with Life Scale* (SLS).^9^
- *Measure of loneliness*: UCLA Loneliness scale.^10^
- *Other measures used as potential covariates: Brief Resilience Scale* (BRS),^11^ *Hospital Anxiety and Depression scale* (HADS),^12^ *EQ‐5D*, a generic quality of life measure with its five dimensions, including pain and mobility,^13^ *Life Orientation Test* (LOT),^14^ *Eysenck Personality Questionnaire* Short Form (EPQ‐SF), *Self‐Esteem Questionnaire* (SEQ).^15^

*The self‐reported stage of illness in PD*,^16^ a modified and previously validated^16^ Hoehn and Yahr scale^17^ for self‐report, was used to characterize the stage of illness in PD at baseline and one year later.

### Statistical Analysis

IBM SPSS v29 for MAC was used for analysis. The significance level was set at 0.05. False discovery rate was used to correct for multiple comparisons.^18^ A *χ* ^2^‐test was performed to determine differences in gender, marital and employment status between groups. Independent‐samples two‐tailed *t*‐tests were used to assess the differences between PD and FSCD in terms of age, disease duration and the differences in resilience (BRS), anxiety and depression (HADS), pain and mobility subdomains EQ‐5D, life orientation (LOT), extroversion and neuroticism (EPQ‐SF) and self‐esteem (SEQ). The measures showing significant differences between groups were then used as covariates in a series of repeated measures ANCOVAs to test the main effects of Time and Group and the Time × Group interaction on the measures of SWB and loneliness in PD and FSCD. $\eta_{p}^{2}$ was used as an effect size measure for the ANCOVA. Paired‐samples two tailed *t*‐tests were used for the planned post‐hoc analysis to assess the effect of time on different measures of SWB/loneliness where appropriate as well as to assess the differences in self‐rated stage of illness in PwPD. Cohen's *d* was used as an effect size measure of the paired‐samples *t*‐test. The post‐hoc power analysis was calculated by the use of online MedCalc software for comparisons of means^19^ and paired samples *t*‐test^20^ (Supporting Information S2).

## Results

Table 1 shows the differences in age, gender, disease duration, mood, personality, and pain and mobility subdomains of the EQ‐5D between PwPD and PwFSCD. There was no difference in self‐reported stage of illness in PwPD at baseline (1.91 ± 1.33) and one year later (2.03 ± 1.04), *t*(52) = −0.75, *P* = 0.203, *d* = −0.11. Disease duration was longer in PwFSCD than in PwPD (*P* < 0.001). People with FSCD reported more pain (*P* = 0.009) and lower self‐esteem than PwPD (*P* = 0.011). Therefore, disease duration, pain and self‐esteem were then used as covariates in the subsequent analysis (Table 2). The effects of Time, Group and the interaction effect Time×Group were not significant for subjective happiness, life satisfaction, or loneliness (Table 2).

**TABLE 1:** Differences in age, gender, disease duration and mood, personality and quality of life measures between people with Parkinson's Disease (PD) and people with focal/segmental craniocervical dystonia (FSCD)

	PD	FSCD	*P‐*value
Age	67.93 ± 8.35	67.03 ± 10.73	0.591
Gender (F:M)	29:24	25:16	0.064
Employment (R:U:Fte:Pte)	30:2:11:10	20:0:7:4	0.062
Marital status (Ma:S:Sep:D:W:Par)	40:3:0:4:6:0	27:4:0:4:6	0.056
Disease duration (years)	7.37 ± 6.14	26.11 ± 15.31	**<0.001**
Brief resilience scale	3.46 ± 0.18	3.49 ± 0.12	0.798
Hospital anxiety and depression score‐anxiety	7.23 ± 0.47	7.87 ± 0.66	0.445
Hospital anxiety and depression score‐depression	5.89 ± 0.37	5.62 ± 0.52	0.658
EQ5D‐mobility	2.31 ± 0.12	1.98 ± 0.15	0.095
EQ5D‐pain/discomfort	2.17 ± 0.11	2.59 ± 0.12	**0.009**
Life orientation total score	14.46 ± 0.47	14.26 ± 0.76	0.817
EPQ‐SF: extraversion	6.15 ± 0.42	5.00 ± 0.54	0.094
EPQ‐SF: neuroticism	5.32 ± 0.36	5.59 ± 0.55	0.660
Self‐esteem questionnaire	21.38 ± 0.49	19.12 ± 0.75	**0.011**

*Note*: The significant ***p*‐values** are written in bold

Abbreviations: EQ5D, Quality of life Scale; EPQ‐SF, Eysenck Personality Questionnaire Short Form; F, females; M, males; R, Retired; U, Unemployed; Fte, Full time employment; Pte, Part time employment; Ma, Married; S, Single; Sep, Separated; D, Divorced; W, Widowed; Par, Having a partner.

**TABLE 2:** Changes in the measures of subjective well‐being and loneliness in people with Parkinson's disease (PD) and people with focal‐segmental craniocervical dystonia (FSCD) over a one‐year period

	PD		FSCD		ANCOVA		
	Time 1	Time 2	Time 1	Time 2	Time	Group	Time × Group
Subjective Happiness Scale	5.21 ± 0.41	5.04 ± 0.45	4.76 ± 0.61	4.59 ± 0.62	*F*(1,77) = 0.47, *p* = 0.495, $\eta_{p}^{2}$ = 0.006	*F*(1,77) = 0.62, *p* = 0.433, $\eta_{p}^{2}$ = 0.008	*F*(1,77) = 0.68, *p* = 0.414, $\eta_{p}^{2}$ = 0.009
State Hope Scale—Pathways	23.14 ± 2.80	20.59 ± 3.81	19.45 ± 2.30	19.77 ± 2.20	*F*(1,76) = 0. 1, *p* = 0.983, $\eta_{p}^{2}$ = 0.001	*F*(1,76) = 0.751, *p* = 0.389, $\eta_{p}^{2}$ = 0.010	*F*(1,76) = 3.08, *p* = 0.145, $\eta_{p}^{2}$ = 0.028
State Hope Scale—Agency	23.97 ± 2.55	20.67 ± 1.81	19.22 ± 2.31	21.00 ± 2.35	*F*(1,71) = 3.28, *p* = 0.074, $\eta_{p}^{2}$ = 0.044	*F*(1,71) = 1.83, *p* = 0.180, $\eta_{p}^{2}$ = 0.025	*F*(1,71) = 13.62, **p < 0.001**, $\eta_{p}^{2}$ = 0.161
Meaning in Life Questionnaire—Presence	19.97 ± 3.51	19.55 ± 2.20	15.12 ± 3.14	19.51 ± 2.35	*F*(1,76) = 17.66, **p < 0.001**, $\eta_{p}^{2}$ = 0.189	*F*(1,76) = 2.68, *p =* 0.111, $\eta_{p}^{2}$ = 0.033	*F F*(1,76) = 12.78, **p < 0.001**, $\eta_{p}^{2}$ = 0.190
Meaning in Life Questionnaire—Search	14.16 ± 3.70	14.86 ± 3.01	22.52 ± 4.11	17.93 ± 4.13	*F*(1,76) = 0.92, *p* = 0.340, $\eta_{p}^{2}$ = 0.012	*F*(1,76) = 6.27, **p = 0.014**, $\eta_{p}^{2}$ = 0.076	*F*(1,76) = 4.12, **p = 0.046**, $\eta_{p}^{2}$ = 0.051
Subjective Life Satisfaction	24.00 ± 3.11	23.68 ± 3.21	21.13 ± 3.61	20.38 ± 3.72	*F*(1,78) = 0.99, *p* = 0.321, $\eta_{p}^{2}$ = 0.013	*F*(1,78) = 0.69, *p* = 0.409, $\eta_{p}^{2}$ = 0.009	*F*(1,78) = 1.38, *p =* 0.244, $\eta_{p}^{2}$ = 0.017
UCLA—Loneliness Scale	34.95 ± 4.51	37.39 ± 4.42	40.13 ± 7.21	54.80 ± 9.45	*F*(1,75) = 2.17, *p* = 0.145, $\eta_{p}^{2}$ = 0.028	*F*(1,75) = 1.81, *p* = 0.183, $\eta_{p}^{2}$ = 0.024	*F*(1,75) = 1.28, *p =* 0.261, $\eta_{p}^{2}$ = 0.017

*Note*: Significant ***p*‐values** are shown in bold.

### Change over Time in Hope

There were no significant effect of Time, Group and the interaction effect Time×Group for *hope pathways*. The effect of Time and Group were also not significant for *hope agency*. However, the interaction effect of Time × Group for *hope agency* was significant (*P* < 0.001). This was due to the drop in hope agency for PwPD over time, *t*(46) = 5.24, *P* < 0.001, *d* = 0.76, compared to the increase in agency for PwFSCD, *t*(32) = −2.34, *P* = 0.026, *d* = −0.41.

### Change over Time in Meaning in Life

While the effect of Time was significant (*P* < 0.001) for *presence of meaning in life*, the effect of Group (*P* = 0.111) was not significant. The interaction of Time × Group was significant (*P* < 0.001):over time *presence of meaning of life* increased for PwFSCD, *t*(34) = −2.22, *P* = 0.021, *d* = −0.41, but did not change for PwPD, *t*(48) = 0.86, *P* = 0.198, *d* = 0.12.

There was no significant effect of Time on *search for meaning in life* (*P =* 0.340), but there was a significant effect of Group (*P* = 0.014) and a significant Time×Group interaction (*P* = 0.046). This was due to decrease of search for meaning in life for PwFSCD over time, *t*(34) = 1.913, *P* = 0.041, *d* = 0.28. The change of search for meaning in life was not significant for PwPD, *t*(48) = −1.46, *P* = 0.151, *d* = −0.21.

## Discussion

There were no changes over the one‐year period for both PwPD and PwFSCD in two measures of SWB—subjective happiness and life satisfaction—as well as in loneliness. While hope agency decreased in PwPD, it increased in PwFSCD over one year. The meaning in life presence score increased for PwFSCD but remained stable in PwPD. By contrast, the meaning in life search score decreased in PwFSCD, and it did not change in PwPD.

### No Longitudinal Changes in Subjective Happiness, Satisfaction with Life and Loneliness in PwPD and PwFSCD

The finding that the levels of happiness, life satisfaction and loneliness tend to remain stable over time is consistent with the previous research.^21^, ^22^, ^23^ In addition to no changes for PwPD, there were also no changes in PwFSCD suggesting short‐term stability in these hedonic and social well‐being domains despite the presence of chronic neurological conditions. These findings are encouraging, as they indicate that the core aspects of hedonic well‐being (subjective happiness), evaluative well‐being (life satisfaction), and social connectedness (loneliness) remain stable in both condition albeit over a short follow‐up period.

### Longitudinal Changes in Hope in PwPD and PwFSCD

While hope pathways—the ability to find ways to achieve goals—remained stable in both Pw PD and PwFSCD, hope agency—the ability to set goals—decreased in PwPD and increased in PwFSCD. Although we did not find change in the self‐reported stage of illness in PD, the decrease in hope agency over one year in PwPD aligns with the progressive nature of the disease as the slow worsening of motor and non‐motor symptoms such as bradykinesia, tremor, apathy, and fatigue along with increasing dependency on others^24^, ^25^ can erode perceived control.^26^ The degenerative nature of PD can challenge and diminish the willpower to pursue goals.^7^ By contrast, the increase in hope agency for PwFSCD suggests that the relatively stable and slow‐progressive nature of FSCD can allow for moments of renewed motivation and suggests that as people continue to live with their chronic symptoms they learn to adapt and can find moments of reclaiming initiative.^5^

### Longitudinal Changes of Meaning in Life in PwPD and PwFSCD

While there were no significant changes in presence or search for meaning in life in PwPD, there was a significant increase in meaning in life presence score and a decrease in meaning in life search score in PwFSCD. The lack of change in meaning in life scores in PwPD can be explained by the fact that PwPD do not passively accept decline; they actively adapt by re‐evaluating and reconfiguring what gives their life purpose and coherence.^27^ This may therefore preserve their sense of meaning so they do not need to search for more purpose than they already have. The meaning in life presence score, which at baseline was lower in PwFSCD compared to PwPD, increased after one year in PwFSCD. This was, as expected, paralleled by a decrease in the meaning in life search score in PwFSCD. Dystonia can impact work, hobbies and relationships all aspects of life related to meaning in life.^28^, ^29^ Our results indicate that PwFSCD continue to search for meaning leading to active coping strategies (eg, finding new purposes) that help maintain or raise their presence of meaning score.

There are several limitations of the study. One year is a relatively brief follow‐up period for PwPD and PwFSCD. Although a longer observation period might have contributed to understanding changes in different aspects of subjective well‐being and loneliness, the natural progression of the diseases could have interfered with the interpretation of the results. Nevertheless, it would be valuable to replicate this study by comparing disease groups over longer periods at multiple assessment points, in order to clarify how different aspects of SWB and loneliness change over time and relate to disease progression. We collected the data by sending the questionnaires and scales to the patients by post. Therefore, we were unable to evaluate the objective motor status of the patients who participated in the study and used only the self‐reported stage of illness, which can correctly reflect the motor stage of PD as previously validated.^16^ No measure of illness severity was used for participants with FSCD.

In conclusion, SWB evaluation and loneliness are generally stable over one year in PwPD and PwFSCD, but there are disease‐specific changes in certain domains of SWB, such as hope and meaning in life. Further longitudinal studies with longer follow‐up periods are needed to further explore the longitudinal changes in PwPD and PwFSCD and their relationship to disease progression.

## Author Roles

(1) Research project: A. Conception, B. Organization, C. Execution; (2) Statistical Analysis: A. Design, B. Execution, C. Review and Critique; (3) Manuscript: A. Writing of the first draft, B. Review and Critique.

S.S.: 1A, 1B, 1C, 2C, 3A, 3B

D.G.: 2A, 2B, 2C, 3B

P.J.: 1C, 3B

K.B.: 1C, 3B

P.L.: 1C, 3B

M.J.: 1A, 1B, 1C, 2A, 2C, 3B

## Disclosures

**Ethical Compliance Statement:** Full ethical approval was granted by Health Research Authority and Health and Care Research Wales Ethics Committee. Full ethics approval for the project was obtained on 2nd February 2018, application number 233401, REC Ref 18/LO/1368. As suggested by the Ethics Committee who reviewed and approved the proposal, the return of the completed booklet acted as implied consent to participate in the study. We confirm that we have read the Journal's position on issues involved in ethical publication and affirm that this work is consistent with those guidelines.

**Funding Sources and Conflicts of Interest:** DG, was partially supported by the Slovenian Research and Innovation Agency (ARIS) project programme P2‐0209 Artificial intelligence and intelligent systems. The authors declare that there are no conflicts of interest relevant to this work.

**Financial Disclosures for the Previous 12 Months:** The authors declare that there are no additional disclosures relevant to this work to report.

## Financial Disclosures and Conflicts of Interest

Author disclosures are available in the Supporting Information.

## Supporting information


**Data S1.** Supporting Information S1

**Data S2.** Supporting Information S2

**Data S3.** COI_Disclosure

## Data Availability

The data that support the findings of this study are available from the corresponding author upon reasonable request.
