# Supplementary material for: Longitudinal Changes in Subjective Well‐Being and Loneliness in Parkinson's Disease and Focal/Segmental Craniocervical Dystonia
Source: Mov Disord Clin Pract. 2026 Aug 3:10.1002/mdc3.70742. Online ahead of print. doi: 10.1002/mdc3.70742 (PMC13434061; doi:10.1002/mdc3.70742)
Supplement: Supplementary file 2 [file MDC3-9999-0-s002.docx]

Supplementary Material 2. Values of the measures of subjective well-being and loneliness, post hoc power (calculated by MedCalc Software) for the comparisons between PD (Parkinson's disease), n = 59, and FSCD (Focal/Segmental Cervical Dystonia), n = 49, groups at Time 1 and Time 2, and post hoc power for the within-group comparisons (Time 1 vs. Time 2).

|  | **Parkinson’s disease** | | | **Focal/Segmental Cervical Dystonia** | | | **Post-Hoc power**  **Between Groups** | |
| --- | --- | --- | --- | --- | --- | --- | --- | --- |
|  | Time 1 | Time 2 | Post-hoc power | Time 1 | Time 2 | Post-Hoc Power | Time 1 | Time 2 |
| **Subjective Happiness Scale** | 5.21±0.41 | 5.04±0.45 | 0.847 | 4.76±0.61 | 4.59±0.62 | 0.479 | 0.990 | 0.988 |
| **State Hope Scale - Pathways** | 23.14±2.80 | 20.59±3.81 | 0.990 | 19.45±2.30 | 19.77±2.20 | 0.001 | 0.990 | 0.001 |
| **State Hope Scale - Agency** | 23.97±2.55 | 20.67±1.81 | 0.990 | 19.22±2.31 | 21.00±2.35 | 0.999 | 0.990 | 0.001 |
| **Meaning in Life**  **Questionnaire - Presence** | 19.97±3.51 | 19.55±2.20 | 0.674 | 15.12±3.14 | 19.51±2.35 | 0.990 | 0.990 | 0.001 |
| **Meaning in Life Questionnaire - Search** | 14.16±3.70 | 14.86±3.01 | 0.990 | 22.52±4.11 | 17.93±4.13 | 0.990 | 0.990 | 0.991 |
| **Subjective Life Satisfaction** | 24.00±3.11 | 23.68±3.21 | 0.589 | 21.13±3.61 | 20.38±3.72 | 0.001 | 0.991 | 0.998 |
| **UCLA -Loneliness Scale** | 34.95±4.51 | 37.39±4.42 | 0.910 | 40.13±7.21 | 54.80±9.45 | 0.990 | 0.889 | 0.990 |
